# The Role of Digital Media in Chronic Disease Self-Management: Protocol for a Multimethod Study of the DISELMA Research Consortium

**DOI:** 10.2196/77811

**Published:** 2026-01-28

**Authors:** Constanze Rossmann, Veronika Karnowski, Julia Metag, Juliana Raupp, Doreen Reifegerste, Claudia Riesmeyer, Nariman Sawalha, Alexandra Lux, Anna-Lena Esser, Rebecca Kammerer, Franca Singh, Natalie Rödel, Janine Brill, Elisabeth Gerling, Annemarie Wiedicke

**Affiliations:** 1 Department of Media and Communication, LMU Munich, Oettingenstr. 67, Munich, 80538, Germany, 49 89 2180 9424; 2 Institute for Media Research, Chemnitz University of Technology, Chemnitz, Germany; 3 Department of Communication, University of Münster, Münster, Germany; 4 Institute for Media and Communication Studies, Freie Universität Berlin, Berlin, Germany; 5 School of Public Health, Bielefeld University, Bielefeld, Germany

**Keywords:** digital media, mHealth, self-management, diabetes, COPD, asthma, ecological model, continued use, network approach, framing approach, mobile health, chronic obstructive pulmonary disease

## Abstract

**Background:**

Chronic diseases, such as type 1 and type 2 diabetes, asthma, and chronic obstructive pulmonary disease , demand long-term treatment and permanent adaptation. One important pillar in coping with these diseases is individuals’ self-management, including support from digital media. Research on their effects confirms their potential. However, it is flawed by theoretical underdevelopment and methodological weaknesses, such as a focus on short-term effects, single digital features, and microlevel studies.

**Objective:**

The research unit (RU) DISELMA (“Digital Media in Chronic Disease Self-Management”) aims to examine the continued use patterns and effects of the digital self-management of chronic diseases, as well as the role of the interpersonal, organizational, and societal levels to gain a comprehensive picture of the individual processes, their contextual embeddedness, and cross-level interactions.

**Methods:**

To fully capture the manifold multilevel influences, the RU comprises 6 individual projects (IPs), each of which conducts several studies. Two projects at the individual level analyze determinants of use, usage patterns, and effects of digital media, combining systematic reviews, experience sampling method studies, focus groups, panel surveys, and content analysis of apps used. Two projects examine the interpersonal context by analyzing the role of health care providers and the diffusion of digital media in informal networks, conducting a scoping review, online surveys with physicians, semistructured interviews, and participant observations of physician-patient dyads, patient focus groups, and interviews with peers. One project aims to analyze the role of organizations within the mobile health market by conducting a content analysis of organizational messages and a survey. Finally, one project analyzes journalistic and social media to gain insight into the discourses about digital chronic disease self-management on the societal level.

**Results:**

The RU received funding approval from the Deutsche Forschungsgemeinschaft (German Research Foundation; grant 456132969) in July 2023, and the 4-year funding period ranges from December 2023 to November 2027. IP1 is currently conducting its systematic reviews and experience sampling method studies, both to be finalized in 2026. IP2 is conducting its systematic review and meta-analysis alongside panel surveys until June 2026. IP3 has completed its online survey with physicians and is currently conducting observations until August 2026. IP4 is conducting its scoping review and peer interviews through 2026, while IP5 is working on its content analysis and survey, and IP6 on its manual content analysis. First publications of the results are expected in 2026.

**Conclusions:**

The results will contribute to the existing research through a theoretically and methodologically comprehensive approach that improves our understanding of the processes within and between all levels. These insights will inform providers of digital health solutions and health care practitioners about users’ needs, advance evidence-based disease self-management programs, and contribute to better coping with chronic diseases, improved well-being of affected individuals, and reduced health care costs.

## Introduction

### Background

As a consequence of both lifestyle changes and a growing number of older people, chronic diseases are the most common and economically significant health problems in industrialized countries [1-3]. Among these, type 1 and 2 diabetes, asthma, and chronic obstructive pulmonary disease (COPD) belong to those chronic diseases with the highest prevalence rates, ranging between 6% and 10% in Germany [4-7]. Given that chronic diseases are prolonged, do not resolve on their own, and are rarely cured completely, they are characterized as lifelong challenges that demand long-term treatment and permanent adaptation [8]. Despite numerous therapeutic options, disease control remains inadequate in most of these conditions [9]. In addition, most chronic diseases are heavily influenced by a patient’s daily life habits, such as dietary behavior, physical activity, smoking, and medication adherence [10], requiring individuals to be responsible for their health. Consequently, self-management is becoming an important pillar in coping with chronic diseases [11].

### Self-Management of Chronic Diseases

The self-management of chronic diseases embraces three different domains: (1) the acquisition of knowledge about one’s condition and its treatment; (2) behavioral management, including both medical adherence and everyday life management (condition management); and (3) emotional management for maintaining adequate psychosocial functioning [12,13]. To facilitate the self-management of chronic diseases, several disease management programs (DMPs) have been developed. They offer a structured and systematized schedule and provide patients with an understanding of their disease and the behavioral skills that they need to master, monitor, and respond to their current health status [14]. In Germany, the statutory health insurance funds offer DMPs for, for example, asthma, COPD, and type 1 and 2 diabetes [15,16]. Ultimately, self-management programs aid in improving health and quality of life by helping patients gain a sense of control over their disease and preparing them for disease management at home [17,18].

However, meta-analyses examining the effects of such traditional chronic disease self-management programs across various chronic diseases show that interventions often have small to medium, unsustainable effects on clinical outcomes and behavior change [19,20]. In the context of diabetes self-management, several reviews and meta-analyses have found small improvements in disease-specific knowledge and clinical parameters [21,22]. Regarding asthma and COPD, some evidence suggests that the frequency of dyspnea is reduced and that adherence to written action plans is enhanced after following self-management programs [23]. However, almost no effects were found for hospital admissions [24,25] and health-related quality of life [24]. Furthermore, studies on both diabetes and respiratory diseases have observed a high chance of relapse, especially after interventions on weight loss, exercising, and smoking cessation [26-28], high dropout rates, and overall low participation [29].

The observation of small benefits, relapse, and low uptake of traditional self-management programs emphasizes the need for improvement, especially regarding the inclusion of emotional management in addition to mere medical management strategies [30,31], tailoring to individual needs and obstacles [32], and offering continuous day-to-day support to combat lack of self-efficacy and motivation, as well as to facilitate engagement in self-management behaviors [26]. In comparison to one-time or short-term self-management programs, interventions with regular and reinforced follow-ups have been substantially more promising [21]. However, traditional face-to-face programs are naturally limited by accessibility constraints and time restrictions [33]. These circumstances underscore the urgent need for innovative interventions that accompany patients through their everyday lives and are tailored to their specific needs—demands that can be achieved using digital media for chronic disease self-management.

### Digital Media in Chronic Disease Self-Management

Over the past decades, digital media have become deeply embedded in people’s everyday lives. Considering the great variety of devices, services, and platforms, digital media allow a broad range of uses, building upon the core assets of multimediality and interactivity, granting communication on the continua from synchronous to asynchronous and interpersonal to mass communication [34,35]. This digitalization has transformed virtually all aspects of our lives and every area of society [36,37]. Even more so, mobile media, which now encompasses a growing proportion of digital media, has enabled ubiquitous access to digital content across all aspects of our lives. Hence, in current media ecologies, users are most often provided with access to mobile, digital, and internet-enabled devices (such as smartphones) throughout their daily lives. These devices allow constant access to a wealth of information, communication, and other services, which users can continuously arrange and rearrange according to their specific needs [38].

Against this background, digital media technologies have become predestined to improve chronic disease self-management and facilitate long-term adherence. This potential is further enhanced through the dedicated use of digital media for health information and services (eHealth) or the use of mobile media for health purposes, such as health care or health promotion (mobile health [mHealth]) [39-41]. Indeed, recent use data have shown that information procurement for health matters is increasingly occurring online and on mobile. According to the Health Information National Trends Survey Germany, the internet is the first port of call for 1 in 5 Germans when seeking health-related information (21%), second only to physicians (69%) [42]. In addition, digital offers are gaining momentum in the diagnosis and therapy of diseases. Almost one-third of patients already use apps to manage their disease or monitor health data [43,44].

Key use cases of eHealth and mHealth include the collection, management, processing, sharing, and storage of patient-reported data and clinical parameters [45,46], also through self-tracking systems. By affording convenient and continuous access to timely personal data and health-related information, individuals living with chronic diseases are encouraged to—quite literally—take matters into their own hands [39,47,48]. Even if this places more responsibility on individuals living with a chronic disease, they are not necessarily on their own. In fact, eHealth and mHealth can facilitate and even increase the frequency of communication between patients and health care providers [39,49,50]. In addition, by integrating technology, physicians can remotely adjust treatment plans, provide the necessary feedback, and conduct regularly scheduled online consultations [51,52]. Increasingly, informal caregivers (ie, relatives) are involved, either by accessing the same digital health system as patients or by using a stand-alone caregiver system. To fulfill their caregiving responsibilities, digital media provide informal caregivers with a platform to continuously learn about the patient’s disease, help manage medical care, and provide practical and emotional social support, both mobile and online [53]. Furthermore, traditional health care providers and informal caregivers have been joined by new players, such as technology companies. These are also involved in chronic disease management through the proliferation of digital health technologies [54]. Thus, disease management becomes a collaborative process between actors on various levels in which individuals with chronic diseases are embedded and actively involved.

With these possibilities, digital health technologies can foster adherence to disease self-management more effectively than conventional health care models. While traditional self-management programs remain anchored in didactic structures, which only teach patients why, how, and when to perform certain self-management behaviors and rely on them to take them into practice [55,56], digital systems accompany individuals with chronic conditions throughout the day. Thus, digital systems can hardly be bypassed, thereby encouraging long-term adherence [57,58]. Ultimately, digital health technologies may present an avenue to strengthen patient empowerment, overcome communication barriers, and enhance treatment adherence. Against this background, health-related digital media have also unsurprisingly become a main priority of political stakeholders in Germany [59,60].

Despite this promising picture, digital technologies not only provide numerous benefits for the self-management of chronic diseases but also bear individual and societal risks associated with their use. Depending on legal standards, the increasing datafication of patients’ health inevitably poses concerns about privacy and misuse of data arising from the conscious or unconscious sharing of data with physicians, service providers, and other third parties [61]. Another vein of skepticism toward digital health technologies lies in the individualization of health. As eHealth and mHealth systems allow remote adjustment of treatment plans, physician visits may occur less frequently, thus actually impairing the development and preservation of a strong and trusting physician-patient relationship [62,63]. Relating thereto, critics argue that the replacement of real-life interactions with digital ones may exacerbate the degree of social isolation and loneliness, which older people living with chronic conditions already regularly experience [64]. Finally, instead of bridging the digital divide and social gaps in health care, digital media carry the risk of widening existing health disparities. Hence, creating equal access to technology and promoting digital literacy among users are priority tasks for society to truly leverage the benefits of digital media in disease self-management [65,66].

### Evidence on the Use and Effects of Digital Media in Chronic Disease Self-Management

Considering that the use of digital media for chronic disease self-management bears both potential and risks, we require profound empirical research on their effects and their conditions of use. Thus far, evidence syntheses provide an inconsistent picture, ranging from positive to limited or no effects on clinical health outcomes (refer to systematic reviews and meta-analyses for various chronic diseases [33,67-72]). For behavioral (eg, dietary behavior or medical adherence) or cognitive outcomes (eg, self-efficacy), the results are also inconsistent [73]. Holmen et al [74] concluded that the effects of research in this context are still too heterogeneous and low in quality to “provide reliable evidence of effects for stakeholders,” which still held true 5 years later [69]. Frequent criticism also mentions a lack of theory and a limitation to single digital media features or apps and short-term effects [67,69,75-78].

Consequently, another branch of research started to examine how and why individuals use digital media for chronic disease self-management to explain the inconsistent evidence for its effects [79,80]. However, again, the explanatory value of most of these studies (although often theory-based) is limited because they are constrained by adoption factors, a rather narrow view of specific tools used, and critical gaps and variability in the measurement of use [81,82]. Only a few studies analyzed continued use [82,83]; however, the multifaceted patterns of everyday life integration are neglected. In a qualitative study with patients with diabetes, Rossmann et al [84] showed that those patients do not use just one specific self-management app; rather, they make use of their entire mobile media ecosystem; for example, they use WhatsApp [Meta] for communication with their caregivers, social media for exchange with other patients, or health websites to seek diabetes-related information. Thus, to fully understand the effects of using digital media in chronic disease self-management, we must look beyond the adoption of single apps and rather consider the continued use of users’ entire digital media ecosystem.

### Need for a Multilevel Perspective

At present, research on the use of digital media for chronic disease self-management has mostly dealt with effects from a microlevel perspective. However, considering the individual level only unduly limits the holistic view necessary to fully grasp digital chronic disease management for two reasons:

(1) a holistic view is necessary to fully understand individual decisions, since individuals living with chronic diseases do not live in a vacuum but are surrounded and potentially influenced by their interpersonal, organizational, and societal contexts. Accordingly, studies provide evidence that the use of digital media for disease self-management highly depends on the recommendations of health care providers [84,85], the support of family members [86], the organizational implications of health data markets [87], and the public discourse in public and social media [88,89]. Thus, these influences must be integrated into research on the use and effects of digital media for chronic disease self-management. (2) Given the contradictory view of the potential and risks of digital media for chronic disease self-management, we will only draw the full picture if we gain an understanding of how professional and personal networks discuss digital chronic disease self-management, how organizational structures evolve in the context of mHealth for chronic disease self-management, and how media discourse develops around this issue.

Hence, the overarching research questions of this research unit (RU) are:

- RQ1: Which role do digital media play in the self-management of chronic diseases?
- RQ2: How is the digital self-management of chronic diseases negotiated on an interpersonal, organizational, and societal level?

### Ecological Model and Objectives

**Figure 1.**
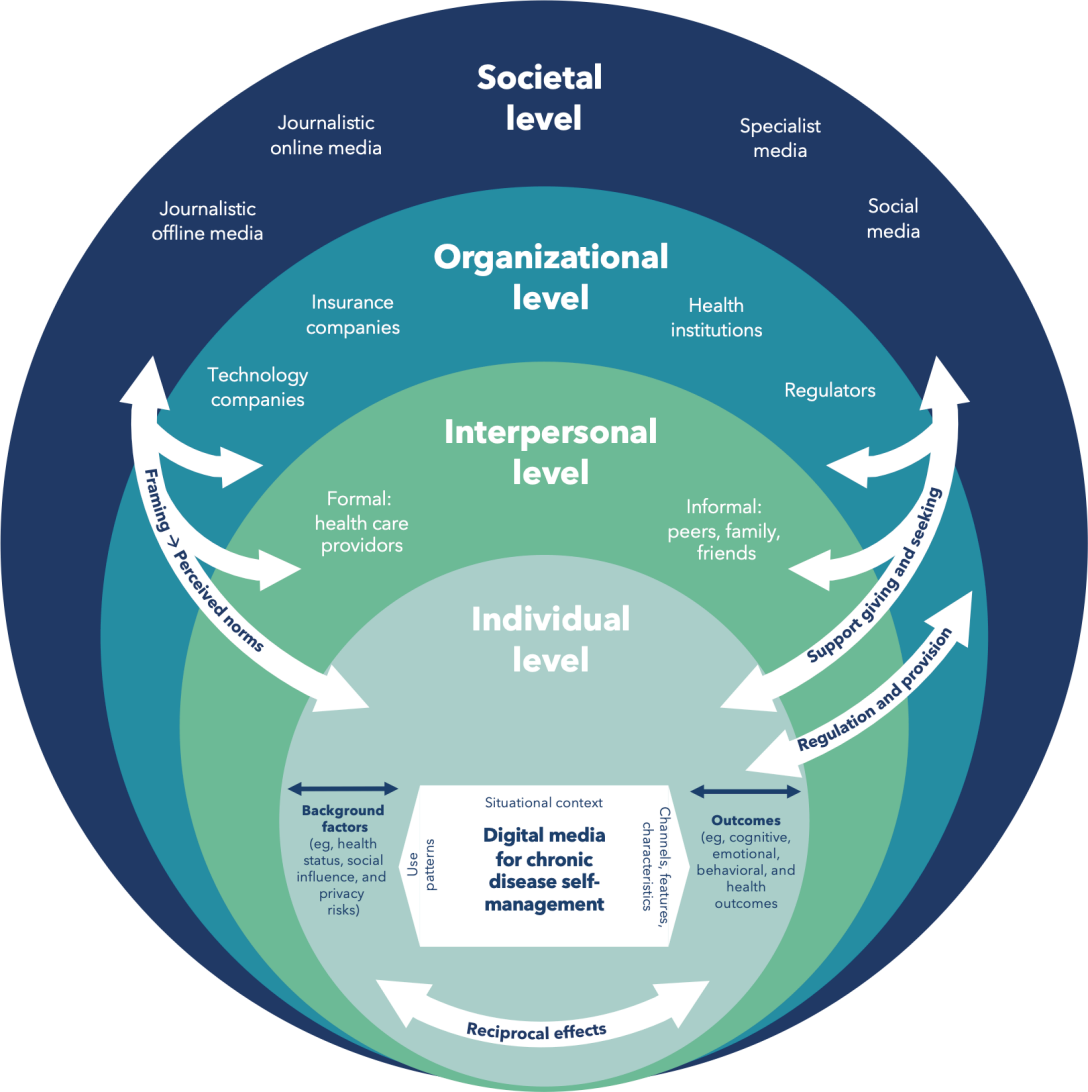
Ecological model of digital chronic disease self-management.

To systemize the various perspectives within our multilevel approach, we adapted the idea of ecological models to digital chronic disease self-management as a basis to derive the specific objectives of the RU. Ecological models have been developed in the behavioral sciences and public health to explain people’s interactions beyond behavioral models that only investigate individual characteristics and their proximal social influences [90]. In addition, an ecological model integrates multiple theories and serves “as a meta-model to ensure that environmental and policy factors are considered in developing comprehensive approaches to studying and intervening on health behaviors” [91]. To structure and integrate these different perspectives into a common framework, we developed an ecological model of digital chronic disease self-management (Figure 1).

The model illustrates interrelations between individual digital media use and its indirect (ie, individual evaluations of behavioral consequences, norm evaluations, and restriction evaluations) as well as its direct context influences at various levels (eg, privacy regulations, cost regulations, and technical developments). In the following, we will describe the factors and interrelations at various levels relevant to the understanding of digital chronic disease management that will be jointly examined in the RU.

#### Individual Level

At the basis of the model are individuals and their manifold digital media use patterns. These patterns are determined by several factors, such as psychological determinants, media characteristics, or situational contexts [85,92-95]. The continued use leads to specific psychological, behavioral, or health-related outcomes evolving over time. Interactions between uses and effects will reinforce each other directly and indirectly via experiences, imposing an impact on future determinants of use and use patterns.

#### Interpersonal Level

The interpersonal level embraces individuals’ social contacts. This includes both formal contacts with health care professionals and informal social contacts, such as peers, friends, and family members [96]. Both groups are related to individuals’ media- and health-related behaviors by mediating knowledge and negotiating how digital media can be used, providing support, or setting norms and values, as postulated in theories of social networks [97,98] and social norms as expectations [99].

#### Organizational Level

Furthermore, communication about eHealth and mHealth has an important organizational dimension. Advertising and organizational messaging are central components of strategic public health communication [100]. How pharmaceutical companies, app developers, health insurance companies, and public health authorities, among others, communicate about digital self-management of chronic conditions influences the perception of digital self-management technologies at both the individual and societal levels.

#### Societal level

Individuals with chronic diseases and their social surroundings are embedded in and influenced by the societal context embracing the political system, economy, cultural norms, and public discourse. Since digitalization, individualization, and the occurrence of specific stressors are (meta-)trends in contemporary societies, they are also topics of public discourse. This means that public discourses about digital chronic disease self-management, as shared in (journalistic) online and offline mass and specialist media, as well as social media, offer frames of an issue associated with evaluations and expectations that are influential for organizations, regulators, health care providers, and individuals [101-103].

This comprehensive perspective not only allows the integration of different levels but also considers cross-level interactions, as apparent in support seeking and giving; formal and informal communication around digital media for chronic disease self-management; the provision and regulation of the app market in relation to individual needs; and framing effects across all levels.

The following objectives emerge from the background and the ecological model of digital chronic disease self-management:

1. To gain an understanding of the continued use patterns of digital media for chronic disease self-management resulting from determinants of use, media characteristics, and situational influences, and the effects of continued use of digital media for chronic disease self-management on cognitive, emotional, behavioral, and health outcomes as well as reciprocal effects.

2. To gain insight into the influences of interpersonal networks on digital chronic disease self-management as imposed by formal (health care providers) and informal (family, friends, and peers) support.

3. To understand the public discourse and framing of digital chronic disease self-management in strategic public health communication (including organizational messaging and corporate media) and in journalistic online and offline media, specialist media, and social media.

4. To gain a conclusive picture of the interrelations among individual, interpersonal, organizational, and societal influences on digital chronic disease self-management, as apparent in framing and perceived norms, support seeking and giving, and regulation and provision.

5. To extend the ecological model of digital chronic disease self-management by theoretically and empirically specifying cross-level relationships.

## Methods

### Overview of Contributions to the Objectives of the Individual Projects

To achieve these objectives, the RU will assemble multimethod studies on the individual, interpersonal, organizational, and societal levels, which are structured into 6 individual projects (IPs). These 6 IPs are conducted by 6 different teams, located at 5 different universities in Germany. Each of the IPs again combines several studies and methods to address the specific research objectives.

Two IPs are located at the individual level and analyze the determinants of continued use, usage patterns, and effects of digital media for the self-management of chronic diseases (objective 1). Specifically, these projects aim to understand the continued use of mobile media in chronic disease self-management by mapping usage situations and their situational context (IP1: use). Furthermore, they aim to understand the effects of use by integrating both a long-term perspective and heterogeneous use patterns (IP2: effects).

Three IPs aim to examine digital chronic disease self-management at the interpersonal and organizational levels (objective 2) by analyzing the role of health care providers within their institutional contexts, the informal social network, and the organizational communication context. Specifically, by transferring the actor-structure-dynamics framework [97] to the health sector, IP3 (health care) aims to understand the relationship between health care providers and patients when adopting and integrating digital media into everyday life. While this project focuses on the professional context, IP4 (peers) studies the influence of informal social contacts and related interpersonal communication on the acceptance of chronic disease–related digital media use. The third project, located at the organizational level, examines organizations’ communication strategies about the use of digital media for the self-management of chronic diseases, contributing to an understanding of the organizational and institutional contexts of digital media use (IP5: organizations).

At the societal level (objective 3), IP6 (reporting) analyzes how digital media use for chronic disease self-management is represented in journalistic and social media discourses. Drawing on framing theory and news value theory, this project focuses on how the topic is constructed, which actors and frames dominate, and how scientific evidence is portrayed and evaluated in the public sphere.

### Overview of IPs

The RU is funded for 48 months. In accordance with the broad spectrum of theoretical backgrounds, the IPs apply various qualitative and quantitative methods, as well as mixed methods approaches. The following paragraphs describe each IP in more detail. For an overview of the IPs and their studies and timelines, refer to Table 1.

**Table 1.** Overview of studies in each individual project (IP).

Studies and methods	Main objectives	Analysis methods	Timeline (start-end)
IP1^a^: use			
Two systematic reviews of current state of health app use (each for diabetes and asthma/COPD^b^)	To gain a comprehensive overview of the measurement of “use” of mHealth^c^ in the self-management of diabetes and asthma/COPD, respectively, in extant research.	Inductive-deductive content analyses	12/2023-12/2025
Two mixed-methods in-situ studies integrating quantitative surveys, mobile experience sampling studies, and automated observations for diabetes and asthma/COPD	To uncover both person- and situation-level types of mHealth use for diabetes self-management and asthma/COPD self-management.	Multilevel latent class using LatentGOLD 6.0 (Informer Technologies) and multivariate analysis methods in R (The R Foundation).	04/2024-12/2026
Two focus groups to contextualize the findings of our 2 in-situ studies based on participants’ individual and subjective experiences	To contextualize our findings with participants’ subjective experiences and beliefs, and brainstorm open questions in a participatory approach.	Inductive-deductive content analyses	01/2027-09/2027
IP2: effects			
Systematic review and meta-analysis of effect studies on mobile chronic disease self-management	Develop and apply a structured framework to synthesize the status quo of mHealth system components and measured self-management outcomes and to meta-analytically assess effectiveness and moderating factors.	Narrative synthesis techniques, 3-level meta-analytical model, and meta-regressions in R.	12/2023-12/2025
Two 4-wave panel surveys to identify patterns of continued mHealth use, behavioral determinants, and long-term effects on behavioral and health outcomes, as well as reciprocal effects for diabetes and asthma/COPD	Identify patterns of continued mHealth use and explore the transactional effects in self-management.	Latent transition analysis, random-intercept cross-lagged panel model, and multigroup analyses in R.	03/2025-12/2026
Content analysis of the mHealth systems used (based on Study 2) using the developed framework (based on Study 1)	Examine the design and assess the quality of commonly used mHealth systems for self-management in Germany.	Descriptive and comparative analyses in R and integrative analysis with Study 2.	06/2026-05/2027
IP3: health care			
Online survey of physicians about their professional health-related media use, recommendation of digital media for self-management, and perception of the physician-patient relationship	Examine the self-perception of actor constellations, preferred physician-patient communication, mediation strategies, digital media within therapy, and participant recruitment for Studies 2, 3, and 4.	Multivariate analysis methods in R.	06/2024-10/2024
Semistructured interviews with 40 physician-patient dyads	Exploring self and mutual perception of actor constellation, social structures, influencing factors, and dynamics and typology of physician-patient relationship.	Theory-driven approach per MAXQDA (VERBI GmbH).	Wave 1: 05/2025-11/2025 and Wave 2: 05/2026-08/2026
Participant observations of consulting hours between the physician and the patient (Studies 2 and 3 were designed as qualitative panels with 2 waves)	Exploring self and mutual perception of actor constellation, social structures, influencing factors, and dynamics and typology of physicians-patient relationship.	Theory-driven approach per MAXQDA.	Wave 1: 05/2025-11/2025 and Wave 2: 05/2026-08/2026
Two focus groups, each with 4 patients and physicians	Discussion, interpretation, and contextualization of the results to identify further open questions.	Theory-driven approach per MAXQDA.	03/2027-06/2027
IP4: peers			
Scoping review on structure, content, and effects of metacommunication in digital chronic disease self-management	Examine the influence of metacommunication on knowledge, persuasion, adoption, and continued use of digital health apps.	Narrative synthesis techniques.	12/2024-12/2025
Qualitative interviews with ego-centered network maps of people with type 1 and 2 diabetes, or asthma/COPD	Exploration of the functions, content, and processes of metacommunication with informal contacts.	Inductive-deductive content analyses.	03/2025-06/2026
Online survey to identify relevant alternatives, their attributes, and tie attributes and gather knowledge about the content and perceived effects	Structural investigation into health-related ego-centered social networks of people with type 1 and 2 diabetes, or asthma/COPD.	Descriptive and multivariate techniques.	06/2026-06/2027
IP5: organizations			
Organizational analysis: typology of central organizational actors based on their characteristics, relationships, and field position	Development of a comprehensive database for further substudies and insights into the institutional structures of the digital health services sector.	Desktop research, documentary analysis, and organizational network analysis.	03/2024-06/2026
Survey of communication practitioners across sectors on attitudes toward digital media for chronic-disease self-management, with follow-up qualitative interviews	Examine organizational communication practices, networks, and affective and normative orientations toward digital health technologies.	Descriptive statistics and regressions in R and qualitative interview analysis for contextualization.	08/2025-08/2026
Automated and manual content analyses of organizational documents, including websites, press releases, and CSR^d^ reports, on digital self-management of chronic diseases	Map value orientations, patient images, normative expectations, and emotional framing in organizational communication.	Website analysis using Python and LDA^e^ packages in R, CSR reports analyzed in MAXQDA with cluster analysis, and descriptive statistics to describe content analyzed press releases.	03/2024-04/2026
Population survey examining how organizational communication affects perceptions of responsibility for chronic-disease self-management	Assess how organizational messages shape responsibility attributions, emotional responses, and normative expectations in the public.	Descriptive statistics and regressions in R.	02/2026-02/2027
Network analysis of central organizational actors and their social media presence, examining characteristics, relationships, and positions within the digital health field	Develop a comprehensive database and analyze how organizations communicate via social media, including how their interactions and positions shape the digital health communication landscape.	Organizational network analysis using R and descriptive analysis of desktop and documentary research.	04/2026-05/2027
IP6: reporting			
Automated content analyses of the entire coverage of chronic diseases	Investigate how the topic of digital media use for the self-management of chronic diseases is constructed within the coverage and to assess its share.	Automated content analysis using the STM^f^ package in R.	12/2023-02/2026
Manual content analysis to tease out the detailed aspects of the coverage of digital media use for the self-management of chronic diseases	Provide a more detailed understanding of how the use of digital media for chronic disease self-management is portrayed in journalistic and social media coverage.	Descriptive and comparative analyses.	04/2025-09/2027

a IP: individual project.

b COPD: chronic obstructive pulmonary disease.

c mHealth: mobile health.

d CSR: corporate social responsibility.

e LDA: Linear Discriminant Analysis.

f STM: structural topic modeling.

#### IP1: Use

IP1 maps the specific situations of mobile media use in chronic disease self-management by building on 2 systematic reviews (Studies 1 and 3), 2 experience sampling studies (Studies 2 and 4), and reflective focus groups (Study 5).

Studies 1 and 3 map the measurement of continued use of mobile media in the self-management of diabetes and asthma/COPD, respectively. Following the PRISMA (Preferred Reporting Items for Systematic reviews and Meta-Analyses) guidelines [104], both systematic reviews are preregistered [105,106]. The selection of databases covered and development of the search string are handled in parallel with IP2, to allow for comparability. Records are stored in the reference management software Zotero (Digital Scholar) and from there transferred to Rayyan (Rayyan Systems Inc) [107]. Coding follows an inductive-deductive approach.

Studies 2 and 4 investigate both person- and situation-level types of mHealth use for diabetes self-management and asthma/COPD self-management. Both Study 2 [108] and Study 4 [109] have been preregistered. Both studies received ethical approval from Chemnitz University of Technology’s Institutional Review Board (IRB). Study 2 and Study 4 each consist of a prequestionnaire, 6 months with 2 daily in-situ measurements per week for 1 week each month, and a postquestionnaire. This process will lead to an ideal maximum of 84 data points per participant (plus pre- and post-questionnaire), exceeding the recommendations given by [110]. Again, following Park and Yu [110], and adding a healthy amount of oversampling, we aim for a sample of 100 individuals per study. The studies are carried out using SoSci Survey*,* with the signals for the in-situ surveys being delivered via text message. A semirandom sampling scheme with predefined intervals was used for signaling: the first signal of the day was sent between 8 AM and 3 PM, and the second signal between 3 PM and 10 PM. Field time for Study 2 covers June 2025 to November 2025, with 103 diabetics taking part in the study; field time for Study 4 will start in February 2026. Both studies will be analyzed using multilevel latent class analysis in LatentGOLD 6.0 (Informer Technologies) [111,112], thus uncovering both person- and situation-level types of mHealth use for diabetes self-management and asthma/COPD self-management.

Study 5 will contextualize the findings of Studies 2 and 4 with participants’ subjective experiences and beliefs, as well as brainstorm open questions for future research in a participatory approach. We will conduct a final qualitative study consisting of 2 focus groups. Interview guidelines will be developed based on the outcomes of Studies 2 and 4. Eight participants from the in-situ study on asthma/COPD and diabetes will be invited to take part in each group. Both focus groups will be audio- and video-recorded and transcribed. Data analysis will be conducted based on Kuckartz [113] using MAXQDA (VERBI GmbH).

#### IP2: Effects

IP2 investigates long-term use patterns and effects of mobile media in chronic disease self-management through 3 complementary studies.

Study 1 (systematic review and meta-analysis) synthesizes mHealth effect studies in type 1 and type 2 diabetes and asthma, and COPD. Following the *Cochrane Handbook for Systematic Reviews of Interventions* [114] and PRISMA statement [104], the preregistered protocol [115] specifies search, eligibility, and analysis procedures. Record screening is managed in Rayyan [107]. Drawing on scoping searches and concept mapping, we develop an organizing framework that decomposes mHealth systems into core components (devices, channels, targeted behavioral contents, features, characteristics, and theoretical grounding) and classifies addressed self-management outcomes into first- (determinants), second- (behaviors), and third-order (health-related outcomes) levels. We then empirically apply this framework by (1) narratively synthesizing mHealth systems and outcomes with frequency distributions and (2) conducting 3-level meta-analyses [116] to estimate effect sizes (Hedges *g*, 95% CI; significance will be set at *P*<.05) and heterogeneity between and within studies (Q test and multilevel *I*² statistics). Meta-regressions with omnibus tests will be used to assess moderating variables (eg, mHealth components and study and sample characteristics). Analysis will be carried out in R (The R Foundation; metafor [117] and dmetar [118]). Study quality will be assessed with the mixed methods appraisal tool [119].

Study 2 (two 4-wave panel surveys) examines how individuals with diabetes (panel 1) or asthma/COPD (panel 2) in Germany use mHealth and how this influences self-management outcomes over time. Two 4-wave online surveys (3-month lags) will be conducted via SoSci Survey with German-speaking adults (≥18 years and smartphone owners). The preregistration [120] details theoretical considerations and the developed questionnaire and specifies the 3 core objectives and analytical steps: (1) identify continued mHealth use patterns through latent class and latent transition analyses, classified by used devices, channels, and system features and characteristics, as well as intended purposes for supporting different self-management behaviors (as per Study 1). Multinomial logistic regression examines predictors of class membership and transitions (eg, determinants of use, privacy perceptions, and demographics); (2) model paths and transactional effects with cross-sectional (Wave 1; structural equation modeling) and longitudinal data (Wave 1‐4; random intercept cross-lagged panel models) to test relations between self-management determinants, behaviors, and health-related outcomes (as per Study 1); (3) multigroup structural equation modelings and random intercept cross-lagged panel models test whether (reciprocal) relations differ across latent usage patterns. Analyses will be performed in R (poLCA [121], depmixS4 [122], and lavaan [123,124]).

Study 3 (content analysis of mHealth systems) examines the design and quality of mHealth systems most used for self-management, based on usage data from Study 2 (names of used apps and websites). All systems used by ≥5 % of participants will be included. The Study 1 framework guides codebook development. Analyses will be primarily descriptive and comparative (eg, chi-square tests). A quality index derived from meta-analytic evidence will be computed and linked to Study 2 data to test the moderating effects of system effectiveness.

#### IP3: Health Care

IP3 expands the overall project by adopting a multimethod design that takes a process perspective on the physician-patient relationship (formal network) and examines the negotiation and mediation processes involved in the use of health-related digital media for chronic disease self-management in their entirety.

Study 1 conducts an online survey with 203 physicians (diabetologists, nephrologists, and pulmonologists). The questionnaire was developed and programmed using SoSci Survey (field time April 2024 to October 2024). Study 1 provides information on physicians’ use of and attitudes toward professionally relevant health-related digital media, as well as their stance on digital media for the self-management of chronic diseases and the perception of the physician-patient relationship. For this purpose, it includes the technology acceptance model [125], which assesses the perceived usefulness and ease of use of information technologies. To gain insights into the theoretical model of the physician-patient relationship [126] and its potential implementation in practice, it uses a validated scale on the physician-patient relationship (adapted versions of PRA and PRA-D) [127,128]. To capture the challenges and recommendations for digital media in self-management, items were developed based on a qualitative systematic review and a meta-synthesis, identifying key themes that facilitate or hinder the use of mHealth or digital health interventions [129,130], complemented by expert interviews with physicians. The questionnaire is available at Open Science Framework [131]. In addition, the surveyed physicians were recruited for Studies 2-4.

Studies 2 and 3 (semistructured interviews and participant observations, 2 waves) use a qualitative approach to examine actor constellations and social structures within the physician-patient relationship. The overarching goal is to identify patterns that characterize the physician-patient relationship during the process of adopting and integrating health-related digital media for chronic disease self-management into everyday life. Semistructured interviews with 20 physicians and 1-2 chronically ill patients (ie, diabetes, asthma/COPD), designed as physician-patient dyads as the smallest form of a formal network, are conducted (Wave 1: field time from May 2025 to November 2025 and Wave 2: field time from May 2026 to August 2026). The interviews take place separately, allowing physicians and patients to open up. The guide includes questions according to the theoretically driven categories on the use of digital media in a private and professional context, the usability and usefulness of digital technologies, as well as the opportunities and challenges of digital self-management in the health care sector. In addition, it covers aspects such as the confidentiality of information sources, social structures, and the physician-patient relationship, including knowledge transfer and shared decision-making. The guide is designed for both physicians and patients, in corresponding versions. In addition, participant observations of the consultation hours between physician and patient (Study 3) are conducted. Due to the complexity and multilayered nature of the multimethod research design, on-site data collection in the physicians’ offices is necessary. Only in this way are interactions between physician and patient observable, and face-to-face interviews can be conducted. All interviews will be transcribed verbatim, anonymized, and pseudonymized. Data analysis will be conducted using a theory-driven approach with MAXQDA [132].

In Study 4 (focus groups), findings from the previous studies will be discussed, interpreted, and contextualized with physicians and patients. Two focus groups, 1 with 4 physicians and 1 with 4 patients, are planned between March 2027 and June 2027. Recruitment will take place during Studies 2 and 3. We will strive for maximum heterogeneity among the invited participants. The focus groups will be transcribed verbatim, anonymized, and pseudonymized. Data analysis will be conducted using a theory-driven approach with MAXQDA [132].

#### IP4: Peers

IP4 investigates the influence of informal network contacts and the related metacommunication on the everyday life integration of digital media for the self-management of the chronic conditions type 1 and 2 diabetes and asthma/COPD.

Study 1 (scoping review) synthesizes results on the structure, content, and effects of metacommunication with informal network contacts in the context of digital media used for illness self-management, based on existing literature on general health topics. The review will systematically examine the influence of metacommunication (alteri, content, and effects), determining the knowledge, persuasion, adoption, and continued use of health apps. Relevant papers will be searched in the databases Communication and Mass Media Complete, PsycInfo, PubMed, and Google Scholar, the journal JMIR (mHealth and uHealth), and the literature of included studies. Specific eligibility criteria will be based on the population, intervention, comparator, and outcome framework and adjusted to the material (eg, considering specifics such as study design, health topic, and methodology). The included studies will be coded based on quality assessment (using the mixed methods appraisal tool [119]), theories used, and results considering data sources, content, and consequences. Data will be analyzed using narrative synthesis.

In Study 2, qualitative interviews with ego-centered network maps are conducted with individuals with (1) asthma/COPD and (2) diabetes to explore the underlying mechanisms, content of metacommunication, and its effect in more detail (eg, who or what initiated the metacommunication about health-related digital media, such as patient, alteri, or a specific event). Thus, the guiding questions of the qualitative interviews are derived from adoption and appropriation models, as well as the scoping review (refer to “IP4: Peers, Study 1”). Both the content and the (perceived) effects are thereby openly queried and considered in the relationship structures. Supported by structuring questions, the interviewees are asked to report on the course of their vicarious information search (occasion, search, selection, transmission, and effects), taking their relationship to the person concerned into account. In addition, the respondents are asked about the sources of information they used for health information search beyond the informal contacts.

In Study 3, a quantitative survey with ego-centered network maps will be conducted with N=900 individuals living with type 1 or type 2 diabetes, or asthma/COPD, to identify relevant social contacts, their attributes, tie attributes, and to gather knowledge about the content and perceived effects. The ego-centered network analysis comprises 3 steps [133]. In the first step, a so-called name generator is used to identify persons (alteri) from whom the respondent received health information in the last 12 months. In the second step, the characteristics of the alteri (so-called alteri attributes) are assessed, such as their health status, their age, and gender. In the third step, details about the relationships between the ego and the alteri (so-called tie attributes) are recorded. Participants will first be asked to name informal individuals or groups (the alteri) who are related to their health-related digital media knowledge, adoption, and appropriation on a network map. This network map will be implemented with a visualization program, such as GENSI [134], and will provide insights into the social network structure of metacommunication about health-related digital media in the self-management of chronic illnesses.

#### IP5: Organizations

IP5 expands the overall project by examining how organizations across sectors communicate about digital health technologies and chronic disease self-management. The project adopts a multimethod design that traces responsibilities, normative expectations, and affective evaluations across organizational actors, public interfaces, and social media environments.

Study 1 has created a comprehensive database of organizations and categorized the actors according to their functions in the organizational field. In parallel, a systematic analysis of the key organizational messages of the most important organizations in this database is in progress. Based on this, an organizational network analysis is carried out, which maps the positions of the various actor groups and the semantic network between selected actors in the institutional field of the digital health market. Furthermore, a theoretical sample of contacts for the survey is being drawn.

Study 2 comprises an online survey of communications practitioners working in the organizations identified in Study 1. The questionnaire assesses attitudes toward digital media for chronic disease self-management, perceived organizational responsibilities, emotional and normative orientations, and assessments of interorganizational communication networks. Recruitment is based on the contact information gathered in the desk research. Participants willing to engage further are invited to expert interviews exploring communicative practices, organizational strategies, and sector-specific perspectives on mHealth technologies. Survey data are analyzed descriptively and through multivariate models, while interview data are examined qualitatively to contextualize the quantitative findings.

Study 3 combines several content analyses. In-depth content analyses of app providers’ websites and organizational reports (eg, corporate social responsibility and diversity, equality, inclusion reports) of major providers of digital health solutions are conducted with regard to the communication of central values, the portrayal of patients and users, and the attribution of responsibilities. Furthermore, automated and manual content analyses of organizational documents, including websites, press releases, and promotional materials, will be conducted to examine how digital media and chronic disease self-management are communicated across various social sectors. The analysis is guided by established approaches to organizational framing and strategic communication [135], which inform the identification of value orientations, patient images, normative expectations, and affective elements. Automated procedures such as dictionary-based emotion detection, semantic network analysis, and indices of empowerment and responsibilization are combined with qualitative coding. This allows us to capture framing patterns and to map how organizations construct digital self-management in their public communication in terms of responsibility, support, risk, and emotion.

Study 4 consists of a population-level online survey that examines how organizational communication affects public perceptions of chronic-disease self-management and digital health technologies. Respondents view selected organizational materials from Study 3 and provide information on their own exposure to organizational campaigns. The questionnaire assesses perceived responsibility for self-management, emotional reactions to digital health tools, and normative expectations associated with chronic conditions. Items also cover evaluations of empowerment, burden, legitimacy, and trust. Data will be analyzed with multivariate statistical techniques to identify how organizational communication influences responsibility attributions, emotional responses, and perceived norms in the general population.

Study 5 investigates how organizational communication circulates and transforms within social media environments, with a focus on platforms such as TikTok (Beijing Bytedance Technology Ltd) and Instagram (Meta). The analysis includes posts from organizational accounts such as health insurers, governmental institutions, NGOs, and pharmaceutical companies, as well as influencer content that engages with or integrates organizational messages. In addition, user comments beneath posts are collected to capture audience reactions, interpretations, and contestations of organizational communication. Sampling includes platform-specific practices such as remixes, collaborations, and responses to organizational campaigns. Automated extraction of textual and metadata features is combined with qualitative coding to identify framing strategies, normative cues, emotional expressions, and responsibility dynamics. The study examines how organizational communication is amplified, reframed, or challenged through the interaction of institutional actors, creators, platform logics, and user engagement.

#### IP6: Reporting

IP6 comprises 2 complementary studies: an automated content analysis (Study 1) and a manual content analysis (Study 2).

Study 1 (automated content analysis) analyzes media coverage of chronic diseases (ie, diabetes, asthma, and COPD) in leading German media outlets from 2010 to the end of June 2025 (N=27,423). The media articles stem from German print and online newspapers, trade and customer magazines, television programs, Instagram post captions from newspapers, Instagram post captions from health influencers, online news sites, and from 4 health forums dedicated to diabetes, asthma, and COPD. The selection of specific media outlets was based on their reach and relevance in the German media landscape. Thus, newspapers and magazines with high circulation figures, online news platforms and health forums with large audiences and members, and health influencers with high follower counts were included. This diverse selection of media types allows for the analysis of different communication logics, ranging from traditional journalistic reporting to personalized storytelling on social media. To identify topics, we conducted a structural topic modeling in R [136]. From this underlying corpus of public discourse on chronic diseases, a subset of articles explicitly addressing the use of digital media for chronic disease self-management will be extracted.

Study 2 (manual content analysis) analyzes how the use of digital media for chronic disease self-management is portrayed in journalistic and social media coverage in more detail. Key categories of the codebook include covered topics, actors, evaluations of health-related digital media, and news factors such as relevance, controversy, personalization, prominence of actors, reach, and negativity [137]. Frames are coded following Dan and Raupp’s [135] typology of frames in health communication (eg, health severity, medical, consequence, gain, and loss). Furthermore, we code how scientific evidence on digital self-management is represented, both explicitly and implicitly, including references to the certainty and uncertainty of research findings [138]. Before starting the manual coding, coders undergo extensive training, and pretests will be run. Krippendorff alpha is used to assess intercoder reliability. Results from both automated and manual analyses will be integrated to provide a thorough interpretation of how digital self-management of chronic diseases is constructed and contextualized in public discourse.

### Joint Research Activities

The global aim of the RU is the integration of IPs within different levels, as well as across different levels, to gain a conclusive picture of the interrelations among the individual, interpersonal, organizational, and societal levels in the context of digital chronic disease self-management (objective 4) and to extend the ecological model of digital chronic disease self-management by specifying interrelations between the different levels (objective 5). These objectives will be achieved by sharing common concepts, jointly developing measurement instruments, and joint data analysis and integration of results from all IPs within the RU, as will be described in the sections on collaborations within concept groups, bi- and multilateral collaborations, and overall integration.

#### Collaboration Within Concept Groups (CGs)

The RU shares several key concepts guiding the IPs and joint activities. Beyond self-management, these pertain to continued use, the network approach, and framing:

##### Continued Use

A prerequisite for the effective implementation of digital media in chronic disease self-management is that individuals not only adopt this technology for a short time, but also integrate the use of digital media for chronic disease self-management into their everyday lives. Nevertheless, previous research on the effects of digital media in chronic disease self-management is dominated by short-term randomized trials, neglecting aspects of use, and research on the use of digital media for chronic disease self-management is mostly bound to adoption decisions, neglecting the crucial role of continued use and everyday life integration. Furthermore, previous research often misses the fact that users differ in their drivers of use and usage patterns, especially when considering not only single apps but also the entire digital media ecosystem. Therefore, this concept group (CG) focuses on the drivers and patterns of the continued use of digital media ecosystems. This common understanding is especially prevalent in IP1, IP2, IP3, and IP4*,* which work together in the CG (continued use). The IPs in this group examine, with varying foci, the individual use of digital media for chronic disease self-management and assess continued use as part of their IPs. On a theoretical level, the IPs reflect on the patterns of continued use and strive to overcome the limitation of focusing on simple adoption decisions and highlight the circumstantiality of such once-off decisions. To optimize the measurement and produce comparable data, this CG jointly develops and implements measures to assess use. Finally, the CG will jointly interpret (and publish) their data, thus contributing to objectives 1, 4, and 5.

##### Network Approach

Previous research indicates that the role of digital media in chronic disease self-management cannot be fully assessed by considering only individuals living with a chronic disease. In contrast, the individual is embedded in a network of health care providers, family, friends, peers, and organizations that may differently influence the use and effects of digital media for chronic disease self-management, which is evidenced by recommendations to use specific apps, the provision of apps to individuals and health care providers, and seeking and giving social support in interpersonal contacts or via social media. Network theory and empirical network analyses are well established in communication science and have become increasingly important in recent years. Several projects apply a network approach and thus broaden the methodological scope of the RU. Network approaches are especially relevant in IP3, IP4, IP5, and IP6, which consequently collaborate within the CG (network approach). Even if these IPs use different specific theories (eg, action theories, theories of adoption and appropriation of media technologies, and social and semantic network theory) and different multimethodological approaches (eg, online surveys, semistructured interviews, participant observations, focus groups, automated content analyses, and ego-centered network maps), the IPs share a common conceptual ground regarding the role of networks, which shall be fostered through joint discussions of various relevant theories and methodological approaches. In this way, the CG will further integrate findings from network approaches into communication research. Finally, integrated analyses will be conducted to contribute to the role of networks and both theoretical and methodological learnings, thus contributing to objectives 2, 4, and 5.

##### Framing Approach

Beyond personal and organizational networks, public and organizational discourses in journalistic and social media and organizational outlets may affect how individuals deal with digital media in managing their diseases as well. The pertaining IPs (IP5: organizations and IP6: reporting) work together in the CG framing approach*,* which commonly draws on the framing approach to identify and describe these discourses. With framing as a key concept within communication research, this CG outlines its specific relevance for the RU and allows for a critical interrogation of its meaning for this specific field. Given the manifold definitions and applications, as well as disciplinary traditions of framing, the IPs exchange their perspectives on framing, find a common conceptual ground, and exchange measures of frames for the identification of frames in the respective IPs. To provide a broad picture of the framing of digital chronic disease self-management from different perspectives, we will integrate the findings to commonly contribute to objectives 2, 3, 4, and 5.

### Bi- and Multilateral Collaborations Between the IPs

Beyond the key concepts and pertaining CGs, the IPs are interlaced in further bi- and multilateral collaborations resulting from common theoretical or methodological approaches, shared measurements, and objects of investigation. For example, IP1 and IP2 share a common theoretical meaning regarding continued use, use patterns, and the conceptualization of mobile media ecosystems. In addition, methodological connections exist between the systematic reviews conducted in both IPs and the common preparation of survey constructs pertaining to use. On this basis, the results of both IPs shall be integrated through method triangulations, contributing to the improvement of theory on use and effects.

Another example is IP3, which has strong ties with IP4, IP5, and IP6. Being located at the interpersonal level, both IP3 and IP4 refer to network approaches as part of the theoretical background and will integrate their results to provide a full picture of the role of formal and informal networks in digital chronic disease self-management. IP3 and IP5 also share the network approach as their common key concept, the notion of embedded agency, as well as their interest in the relevance of various health organizations for physicians and patients. They collaborate in their preparation of interview guidelines and in joint analyses of the mutual perceptions and expectations of market actors, physicians, and patients. In addition to bilateral collaborations, IP3 will also engage in a multilateral collaboration with IP4 and IP6 to jointly develop specific categories in the codebook of IP6 and questions in the interview guides of IP3 and IP4. Through this*,* the 3 IPs will be able to compare the content of exchanges with content in media coverage and jointly analyze mentions of media content in the exchanges.

### Overall Integration

The global aim of the RU is the integration of the IPs within the different levels, as well as between the different levels, to gain a conclusive picture of the interrelations among the individual, interpersonal, organizational, and societal levels in the context of digital chronic disease self-management (objectives 4 and 5). At this stage, the interrelations between the levels are mostly examined by assessing perceptions; that is, IP2 integrates perceived collective norms of digital chronic disease self-management that are understood as a result of framing in public discourse (IP6: reporting) and by organizations (IP5: organizations). Similarly, IP3 and IP5 assess, among other things, how providers of mHealth services anticipate the expectations of their users (IP1: use and IP2: effects). This enables us to specify potential interdependencies. Accordingly, the theoretical goal of these integrated analyses is to improve the ecological model of digital chronic disease self-management over the course of the 4 years of the RU by theoretically specifying (causal) cross-level relationships (objective 5). Consequently, the results from all IPs will be jointly interpreted in the final phase of Year 4.

### Ethical Considerations

All studies collecting data from humans will adhere to the principle of voluntariness and informed consent to participate in the study and to data storage and data publication provided in advance. Studies working with external access panel providers will ensure that the panel provider fulfills the quality criteria with regard to adherence to data protection and ethical rules. In any case, data containing personal information (eg, qualitative interviews) will be stored only as long as necessary and anonymized as soon as possible during the research process to ensure data protection. No datasets with personal information will be shared in open science repositories. Within the framework of a data management plan, binding agreements on the rights of use of the data and materials, their storage locations, and naming conventions will be made.

In addition, each IP will seek IRB approval for each of their studies with human participants. Since each IP conducts multiple studies, many of which build on the results of previously conducted studies, ethical approvals will be obtained in a stepwise process, shortly before the pertaining studies are about to start their fieldwork. Up to now, ethical approvals have been obtained for the following subprojects.

#### IP1: Use

Study 2 (experience sampling method [ESM] study on diabetes), ethical approval granted by the IRB of Chemnitz University of Technology on May 28, 2025 (approval no 101751518); Study 4 (ESM study on asthma/COPD), ethical approval granted by the IRB of Chemnitz University of Technology on November 14, 2025 (extension to approval no 101751518).

#### IP2: Effects

Study 1 (systematic review and meta-analysis), ethical approval granted by the IRB of the Department of Media and Communication, LMU Munich on May 22, 2024 (approval no 2024‐11); Study 2 (panel survey), ethical approval granted by the IRB of the Department of Media and Communication, LMU Munich on May 20, 2025 (approval no 2025‐11).

#### IP3: Health Care

Studies 1-4 (online survey, semistructured interviews, participant observations, and focus groups), approval granted by the IRB of the Faculty of Social Sciences, LMU Munich, on December 6, 2022 (approval no GZ22-05).

#### IP4: Peers

Study 2 (qualitative ego-centered interviews with network maps), approval granted by the IRB at Bielefeld University on November 27, 2024 (approval no EUB-2024‐328).

#### IP5: Organizations

Study 2 (interviews with communication practitioners), approval for quantitative interviews granted on September 29, 2025 (approval no ZEA24041), approval for qualitative interviews granted on February 10, 2025 (approval no ZEA25027), both by the IRB at Freie Universität Berlin.

#### IP6: Reporting

Study 1 (automatic content analysis) and Study 2 (manual content analysis), approval granted by the IRB of the Department of Communication, University of Münster, on May 2, 2024.

For details on ethical aspects per study, refer to Table 2.

**Table 2.** Detailed description of ethical aspects per study.

Study	Human participants	Informed consent, opt-out option	Anonymization	Compensation	No identification in supplements	Ethical approval obtained (approval no)
IP1:^a^ Study 1	—^b^	—	—	—	—	—
IP1: Study 2	Yes	Yes	Yes	Participants taking part in at least 50% of the in-situ surveys receive 75 EUR (a currency exchange rate of 1 EUR=US $1.04 to US $1.17 is applicable).	Yes	101751518
IP1: Study 3	—	—	—	—	—	—
IP1: Study 4	Yes	Yes	Yes	Participants taking part in at least 50% of the in-situ surveys receive 75 EUR	Yes	Extension to 101751518
IP1: Study 5	Yes	Yes	Yes	25 EUR	Yes	Not yet submitted
IP2: Study 1	—	—	—	—	Yes	November 2024
IP2: Study 2	Yes	Yes	Yes	Wave 1: 2.50 EUR, Waves 2 and 3: 4 EUR, and Wave 4: 7 EUR	Yes	November 2025
IP2: Study 3	—	—	—	—	Yes	TBD^c^
IP3: Study 1	Yes	Yes	Yes	100 EUR (cash, voucher, or donation)	Yes	GZ22-05
IP3: Study 2 and 3	Yes	Yes	Yes	Physicians: 400 EUR for both waves and patients: 300 EUR for both waves	Yes	GZ22-05
IP3: Study 4	Yes	Yes	Yes	100 EUR	Yes	GZ22-05
IP4: Study 1	—	—	—	—	—	—
IP4: Study 2	Yes	Yes	Yes	100 EUR	Yes	2024‐328
IP4: Study 3	Yes	Yes	Yes	TBD^c^	Yes	Not yet submitted
IP5: Study 1	—	—	—	—	—	—
IP5: Study 2	Yes	Yes	Yes	None^d^	Yes	Survey: ZEA25027 and interview: ZEA24041
IP5: Study 3	—	—	—	—	—	—
IP5: Study 4	Yes	Yes	Yes	None^d^	Yes	Not yet submitted
IP5: Study 5	—	—	Yes	None^d^	Yes	—
IP6: Studies 1 and 2	—	—	Names of health influencers will be anonymized in datasets	—	Yes	—

a IP: individual project.

b Not applicable.

c TBD: to be determined.

d No compensation provided.

## Results

### General Status of the RU

The project was submitted to the Deutsche Forschungsgemeinschaft in a 2-stage process: the first-stage proposal was submitted in July 2020, reviews and the call to submit the full proposal were obtained in January 2022. Thereafter, we submitted the full proposal in December 2022, orally presented it to the reviewer committee in April 2023, and finally received the funding acknowledgment in July 2023, together with reviews for each of the IPs (refer to Peer Review Report 1 for the reviews in German). After a preparation process (implementation of the projects at the departments, hiring of PhD candidates), the RU started its 4-year funding period in December 2023.

### Status of the IPs

#### IP1: Use

IP1 has finalized the systematic review on continued use of digital media in diabetes self-management (Study 1 of the IP) and is currently working on finishing the systematic review on continued use of digital media in asthma/COPD self-management (Study 3 of the IP). In addition, Study 2, the ESM study on continued use of mobile media in diabetes self-management, is currently being conducted. The last of 6 waves of data collection will be finished by November 2025, and data analysis using multilevel latent class analysis, following the 3-step procedure proposed by Lukočienė et al [139], using LatentGOLD 6.0 [111,112] will start. At the same time, preparations for Study 4 (mirroring the procedures for Study 2) have started, and field time for this study will start in February 2026. Study 5, two focus groups with participants from Studies 2 and 4 to contextualize the results, is planned for early 2027.

#### IP2: Effects

IP2 is currently conducting a systematic review and meta-analysis of studies on mobile chronic disease self-management (Study 1). The planning phase and literature search took place from December 2023 to January 2024, yielding 4852 records. Abstract and full-text screening was conducted from February 2024 to August 2024, narrowing the selection to 202 studies for review. A codebook has been developed, and data extraction is underway. R scripts for data analysis are being prepared. Completion of Study 1 is planned for December 2025.

As of March 2025 to June 2025, we prepared the questionnaire, preregistration, and IRB application for the 4-wave panel surveys (Study 2). Field time for both surveys is scheduled to take place from June 2025 to March 2026, with a time lag of 3 months between each wave. Data collection for Waves 1 and 2 has been completed, resulting in a sample size of n=2171 for diabetes and n=2438 for asthma/COPD in Wave 1, and n=1446 for diabetes and n=1531 for asthma/COPD in Wave 2. The next immediate steps include data cleaning and preparation for Waves 3 and 4, followed by the implementation of cross-sectional and longitudinal analytical models (October 2025 to December 2026).

In parallel, the content analysis (Study 3) will be prepared and conducted over a period from June 2025 to March 2027. This will involve extracting and persisting data on commonly used mHealth systems identified over the course of Study 2. Following the completion of these studies, integrative data analysis will be performed from March to May 2027.

#### IP3: Health Care

IP3 conducted an online survey with physicians from June to October 2024 to analyze their professional use of, perceptions of, and attitudes toward digital media for the self-management of chronic diseases (Study 1), as well as their perception of the physician-patient relationship and their recommendation behavior regarding digital media for chronic disease self-management [131]. Diabetologists, nephrologists, and pulmonologists (N=2808) were recruited via the National Association of Statutory Health Insurance Physicians (KBV) database; n=203 participated in the survey. Physicians were invited by mail, followed by a reminder letter and email. As an incentive, participants could either donate €100 (approximately US $110) to a charity or receive it as a voucher. Data were analyzed using R.

Furthermore, IP3 is conducting Studies 2 and 3. Semistructured interviews and participant observations of 40 physician-patient dyads will be conducted from May to November 2025 (Wave 1) and May to August 2026 (Wave 2) to examine the physician-patient relationship (including comparisons of physicians’ and patients’ self- and mutual perceptions), as well as the recommendation, adoption, and integration of digital media for chronic disease self-management into everyday life. The data will be transcribed verbatim, anonymized, and pseudonymized. The data analysis is based on a theory-driven approach and uses MAXQDA. The coding system is currently being pretested. Coding will start after the data collection of Wave 1. Two focus groups (Study 4) will be conducted between March and June 2027 to contextualize the results.

#### IP4: Peers

First, IP4 conducted a scoping review to analyze the structure of informal network contacts and their role in the use of digital media for the self-management of chronic diseases (Study 1). The preparation and literature search resulted in n=975 hits. After the removal of duplicates, a title and abstract screening was carried out and completed in April 2024. A full-text screening of n=62 identified sources, and the preparation of data analysis is currently ongoing and planned to be completed by December 2025. In addition, IP4 conducted qualitative interviews with peers (Study 2). The corresponding ethics application for the study was approved in November 2024, and the study was carried out from March to September 2025. Study 3, a quantitative survey of peers, is scheduled for 2026. Integrative analysis of qualitative and quantitative data will be carried out in 2027.

#### IP5: Organizations

After completion of Study 1, Study 2 has entered the data collection phase; around 1800 communications practitioners from a broad range of health-sector organizations were identified and contacted, and the survey will remain in the field until February 2026, with first descriptive examinations planned for the end of 2025. Study 3 has completed the compilation and analysis of organizational communication materials. A corpus of approximately 250 organizational websites was crawled, from which keyword and length filters produced around 1000 relevant documents. Complementing this, a focused press release corpus of n=146 items from 38 organizations has been analyzed, with reliability checks completed. Across both datasets, recurring patterns appear in responsibility attributions, value orientations, emotional expressions, and representations of patients in communication on digital self-management. These materials form the empirical foundation for the stimulus development in Study 4, for which questionnaire construction is currently in progress, and fieldwork is scheduled for the middle of 2026. Study 5 has not yet commenced; sampling strategies for organizational posts and user comments on major social media platforms, as well as the technical framework for extraction and coding, are currently being discussed.

#### IP6: Reporting

IP6 has created and validated 2 search strings: 1 to identify journalistic articles covering chronic diseases and another to capture articles specifically addressing the use of digital media for chronic disease self-management. Using these search strings, the main corpus has been compiled, consisting of n=27,423 media articles from German print and online newspapers, trade and customer magazines, television programs, Instagram post captions from newspapers, Instagram post captions from health influencers, online news sites, and 4 health forums dedicated to diabetes, asthma, and COPD. Media articles in this corpus were published between January 2010 and June 2025. Compiling media articles from these various sources was carried out between April 2024 and May 2025. In June 2025, the structural topic modeling was calculated. In total, researchers identified 14 topics within the overall media coverage on chronic diseases: forum discussions on glucose monitoring, lifestyle, cardiovascular diseases, scientific research, health insurance companies, asthma and COPD, diabetes management among adolescents, comorbidities and complications of diabetes, diet and weight loss, food and nutrition, digital diabetes self-management, diabetes, obesity and weight-loss injections, and family. Notably, digital self-management only appears as a distinct topic in the context of diabetes (n=1222), suggesting that discussions of digital health technologies are currently concentrated within diabetes-related reporting, while asthma and COPD receive little attention in this regard. As of November 2025, IP6 has begun pretesting the codebook for the manual content analysis, which will further explore the framing of digital self-management, the representation of scientific evidence, and the role of news factors. Relevant articles will be extracted from the main corpus using the second validated search string, focusing on digital self-management.

## Discussion

### Principal Findings

By bringing together the results from the IPs and collaborating in bi- and multilateral constellations and within the CGs, the RU will be able to jointly develop best practices in measurement, share data and results, and interpret them jointly. Moreover, this collaborative venture will contribute not only to the objectives formulated in each IP but also to common objectives beyond the individual scopes (as outlined in the “Ecological Model and Objectives” section). Finally, the results of all IPs will be integrated to contribute to the overarching objectives of the RU. The expected results will have both scientific and practical implications.

Regarding scientific contributions, four aspects are worth mentioning: (1) both the independent and the integrated results will contribute to improving the theoretical multilevel understanding of digital chronic disease self-management at the individual, interpersonal, organizational, and societal levels and between the levels [84-89]. The IPs mainly operate on one level but are closely related via perceived influences from other levels. By integrating the results from different levels, the results from this stage of the RU will allow for specification of the interrelations between the different levels; (2) the relevance and application spectrum of our results for digital chronic disease self-management have implications beyond this field of research. Disease self-management is closely related to coping, which, in a broader sense and as described in the transactional stress model [140], embraces problem- and emotion-focused strategies to overcome stressors beyond disease-related stressors. Thus, by analyzing coping in this context and applying a multilevel perspective, our results will also contribute to the field of media-related coping research, especially with regard to a closer understanding of problem-focused strategies and the recognition of a situational perspective that has been mostly neglected in coping research [141,142]; (3) beyond coping, many communication phenomena*,* which are usually examined solely with an individual focus or a societal focus will benefit from a multilevel perspective. Sallis and Owen [91] indicated that despite their strengths, ecological models generally are characterized by a “lack of specificity about the most important hypothesized influences” and “the lack of information about how the broader levels of influence operate or how variables interact across levels.” Thus, this project will contribute to previous research in this field by proposing theory-based interrelations and specifying these interrelations relevant to other fields of communication research; (4) the results of this RU will also contribute to methodological development. This refers not only to commonly developed and tested measurement scales (eg, measurement of use within the mobile media ecosystem) but also to insights into experience sampling research, computational methods, and long-term panel research, as well as qualitative and quantitative network analyses.

Moreover, the results of this RU will have several practical implications: (1) the results will provide insights into the existing app market, the continued use of the entire digital media ecosystem by individuals living with chronic diseases [82-84], their effects [33,67-72], and the relevant communication strategies of health care providers, peers, and organizations [96-100], as well as the public discourse on this issue [101-103]. With this, the results will be highly relevant for app developers and health care providers by providing insights into users’ needs and expectations, effective strategies, and the surrounding discourse [100]; (2) furthermore, the results can inform the improvement and advancement of evidence-based disease self-management programs [15,16]. As outlined in the “Introduction” section, traditional disease self-management is often ineffective [19-29]. Thus, by providing evidence on the mechanisms of effective digital disease self-management programs and their surrounding influences, DMPs can be improved; (3) in the long term, this will potentially contribute to better coping with chronic diseases, thus improving the well-being of many people living with chronic diseases; and (4) on a larger scale, this may also be a small but important piece of the puzzle to reduce health care costs in the future.

### Limitations

Our ecological model is by no means exhaustive; it does not include all factors potentially relevant to the interplay of digital media and chronic disease self-management. For example, on the societal level, cultural and political factors may also play a role. However, their influences emerge as part of media discourse or within institutional and organizational structures, and the structural influences on health care providers become evident in formal interpersonal contexts. Thus, our proposed ecological model and the work of the RU will still provide a concise framework to structure and assess the communication processes most relevant to an integrative understanding of digital chronic disease self-management.

### Comparison With Previous Work

Previous research on the effects of digital chronic disease self-management reveals some evidence for its potential [33,67-72] but is sometimes flawed by theoretical underdevelopment and methodological weaknesses, such as the focus on short-term effects, single digital features, and microlevel studies lacking external validity [67,69,75-78]. In addition, it lacks a comprehensive perspective reaching beyond the individual level [74].

Therefore, this RU contributes to existing research by proposing an ecological model of digital chronic disease self-management, integrating different communication perspectives and levels of analysis as well as their interactions, and examining the continued use patterns and effects of digital chronic disease self-management, as well as the role of the interpersonal, organizational, and societal levels, to gain a comprehensive picture of the individual processes, surrounding mechanisms, and cross-level interactions.

### Conclusions

By the end of the project, the results will contribute to the existing research on digital self-management through a theoretically and methodologically comprehensive approach, uniquely situated in communication studies, to improve the understanding of the processes at and between all levels. The results in this context will also allow for conclusions to be drawn about the broader interplay of digital media and coping with modern stressors.

## Supplementary material

10.2196/77811 Peer Review Report 1 Peer review report by the Deutsche Forschungsgemeinschaft (DFG) / German Research Foundation.

## Abbreviations

CG: concept group
COPD: chronic obstructive pulmonary disease
DMP: disease management program
ESM: experience sampling method
IP: individual project
IRB: Institutional Review Board
mHealth: mobile health
PRISMA: Preferred Reporting Items for Systematic reviews and Meta-Analyses
RU: research unit
