# Supplementary material for: The Role of Digital Media in Chronic Disease Self-Management: Protocol for a Multimethod Study of the DISELMA Research Consortium
Source: JMIR Res Protoc. 2026 Jan 28;15:e77811. doi: 10.2196/77811 (PMC12850044; doi:10.2196/77811)
Supplement: Peer Review Report 1 [file resprot-v15-e77811-s001.pdf]

Stellungnahmen zum Antrag

Forschungsgruppe: Digitale Medien im Selbstmanagement chronischer Erkrankungen (DISELMA)“ (FOR 5303)
Projekt: „Koordinationsfonds“ (RO 3067/10-1)

Antragstellende Person: Frau Professorin Dr. Constanze Rossmann, München

---

Stellungnahme der Begutachtungsgruppe:

Der Antrag bringt vorbildlich zum Ausdruck, wie intensiv die Antragstellenden in der Vorbereitung des Antrags zusammengearbeitet haben und welch enge Verzahnung sie in ihren Projekten auf verschiedenen Ebenen planen. Das Ganze ist eindeutig mehr als die Summe seiner Teile. Besonders sind die vier Concept Groups zu erwähnen, in denen zentrale Themen bzw. Konzepte vertiefend diskutiert werden. Diese bilden den Kern der gemeinsamen Theoriearbeit. Die theoretischen Konzepte sind für viele der Einzelprojekte relevant und bilden einen die Gruppe integrierenden gemeinsamen Theoriebezug. Das Verständnis der Konzepte sollte über das im Antrag bereits dokumentierte Maß hinaus vereinheitlicht werden. Hervorzuheben als Modus der Arbeit an einem gemeinsamen Ziel ist auch die kollektive Entwicklung der Instrumente. Der Perspektiven-Reichtum ist beeindruckend und vielversprechend. Der Rahmenantrag ist sehr sorgfältig gearbeitet. Die Relevanz, der Forschungsstand und Forschungslücken sowie die sich daraus ergebende Fragestellungen und Ziele (auch für die zweite Phase der Forschungsgruppe) werden überzeugend entwickelt. Die Begriffsarbeit ist sauber, das Repertoire an Theorien ist vielfältig und für die jeweiligen Fragestellungen jeweils angemessen. Die breit angelegten quantitativen und qualitativen Methoden (inklusive einer vierwelligen Panel-Studie, scraping und automatisierter Inhaltsanalyse, Interviews, Experimenten, Logfile-Analysen, mobile experience sampling) sind im Wesentlichen geeignet, die Fragen zu beantworten. Das Projekt wird als sinnvolles Gesamtwerk einander ergänzender Perspektiven auf verschiedenen Ebenen erklärt, die Antragstellenden entsprechend als Expertinnen und Experten für diese sich ergänzenden Perspektiven. Das Modell illustriert die Verbindung von verschiedenen Ebenen und bettet die auf der Mikro-Ebene angesiedelte Frage nach dem Selbstmanagement nachvollziehbar in verschiedene große Kontexte ein. Im Mikro-Ebenen-Kern wird ein wechselseitiger Einfluss von individuellen Hintergrundfaktoren wie Gesundheit und Privatheitseinstellungen einerseits und Outcomes wie kognitive, konative und emotionale Effekten andererseits dargestellt. Die Ebenen werden verbunden durch Einflusspfeile für erstens Framing und Normenwahrnehmung, zweitens für Unterstützung und drittens für Regulierung und Bereitstellung. Das Arbeitsprogramm ist übersichtlich dargestellt, wobei hier der Schwerpunkt auf den geteilten Theoriekonzepten, den Kooperationen und dem zeitlichen Ablauf liegt. Als besondere Stärken des Projekts sieht die Forschungsgruppe neben den erwähnten Qualitäten auch die Verknüpfung der Ebenen und die Verteilung der Projekte über die Ebenen bzw. auf die Verbindungen dazwischen.

Die Organisation des Projektes ist gut durchdacht, die Fragestellungen sind von hoher Relevanz. Die Begutachtungsgruppe empfiehlt die Förderung im beantragten Umfang – lediglich die beantragten Mittel für Geräte und Software in Höhe von 4.000 Euro sollten gestrichen, die Mittel für Publikationen auf 3.000 Euro gekürzt und die Mittel für Third-party contracts in Höhe von 6.000 Euro sollten gestrichen werden.

Stellungnahmen zum Antrag

Forschungsgruppe: Digitale Medien im Selbstmanagement chronischer Erkrankungen (DISELMA)“ (FOR 5303)
Projekt: „TP1: Nutzungsnischen mobiler Medien im Selbstmanagement chronischer Krankheiten“ (KA 3121/6-1)

Antragstellende Person: Frau Professorin Dr. Veronika Karnowski, Chemnitz

---

Stellungnahme der Begutachtungsgruppe:

Es handelt sich um ein sehr relevantes und spannendes Projekt. Die Relevanz zur Erforschung digitaler Ökosysteme ist eindeutig gegeben, wie aus den bestehenden Forschungslücken (heterogene Studien, geringe Studienqualität, unzureichende theoretische Fundierung, Begrenzung auf einzelne Medienfeatures oder Apps) schlüssig hervorgeht. Der empirische und theoretische Beitrag ist somit klar begründet – der Fokus auf die gesamte digitale Mediennutzung (digital media ecosystem) im Sinne eines Nischenansatzes stellt einen innovativen und relevanten Beitrag für den akademischen Wissensgewinn dar. Das Projekt beruht auf der völlig berechtigten Annahme, dass Mediennutzer/innen heute immer und überall über elektronische Medien mit unzähligen Informations- und Unterhaltungsangeboten in Verbindung stehen (können) sowie über ebenso viele Möglichkeiten der (computerbasierten) interpersonellen Kommunikation verfügen. Diese besondere (und sich auch erst innerhalb der letzten 20 Jahre etablierende) Situation führt zu neuen Formen der Auswahl, der Nutzung, vor allem aber auch der Verarbeitung und damit auch der Wirkung von Medien(-angeboten) und hat entsprechend in der Kommunikationswissenschaft auch zu neuen Theorieansätzen geführt. Die geplante bilaterale Zusammenarbeit ist außerordentlich überzeugend dargestellt.
Die Antragstellerin gilt als ausgewiesene Expertin des Fachgebietes und ist daher im höchsten Maße dazu geeignet, das Projekt erfolgreich umzusetzen. Sie ist eine bestens ausgewiesene und sehr deutlich international agierende Forscherin.
Im Ergebnis hält die Begutachtungsgruppe das Vorhaben für exzellent und empfiehlt die Förderung im beantragten Umfang.

Stellungnahmen zum Antrag

Forschungsgruppe: Digitale Medien im Selbstmanagement chronischer Erkrankungen (DISELMA)“ (FOR 5303)
Projekt: „Einfluss der Nutzung mobiler Medien im Selbstmanagement chronischer Erkrankungen (TP2: Wirkung)“ (RO 3067/9-1)

Antragstellende Person: Frau Professorin Dr. Constanze Rossmann, München

---

Stellungnahme der Begutachtungsgruppe:

Das Projekt stellt das Kernmodul der Forschungsgruppe dar: Es will das Spektrum aller relevanten Kommunikations- und Informationskanäle, Funktionen und Charakteristika im Zusammenhang mit Diabetes und Asthma/COPD systematisieren, längerfristige Nutzungsmuster identifizieren sowie ein umfassendes Wirkungsmodell entwickeln und empirisch analysieren. Die Darstellung des Theorie- und Forschungsstandes, zu dem die Antragstellerin einen erheblichen Anteil selbst beigetragen hat, ist makellos und wird in ein umfassendes und überzeugendes „Process model of mHealth use and its effects on chronic disease self-management“ (Abbildung 1) überführt.
Das Projekt sieht drei empirische Teilstudien vor: In Studie 1 ist eine Meta-Analyse aller relevanten Wirkungsstudien zu mHealth im Themenfeld (Diabetes und Asthma/COPD) geplant. Eine solche Meta-Analyse ist ein beeindruckendes und extrem komplexes Unterfangen, weshalb es gut ist, dass sie auf einer Vorstudie von Sawalha & Rossmann (2022) basiert. Studie 2 sieht zwei Online-Panelbefragungen mit jeweils vier Wellen und fünf Monaten Abstand (Feldzeit 16 Monate) für beide chronischen Krankheiten vor. Der Zeitabstand zwischen den Wellen wird mit der dynamischen Entwicklung digitaler Medien und möglichen dynamischen Veränderungen im Nutzungsverhalten begründet. Auch die Sorge, ob Online-Panel-Anbieter überhaupt in der Lage sind, in derart spezifischen Zielgruppen (erwachsene Smartphone-Nutzende mit Diabetes oder Asthma/COPD) jeweils n=2.000 Teilnehmende realisieren, wird dank eines Angebots von INNOFACT entschärft. Studie 3 umfasst eine Inhaltsanalyse zur öffentlichen Darstellung und Bewertung der Spezifika aller mHealth-Tools und -Anwendungen, die von den Panel-Teilnehmenden innerhalb des gesamten Ökosystems mobiler Medien genutzt werden. Sie wird damit eine Daten- und Erkenntnisquelle für alle anderen Projekte darstellen, sofern sich diese auf einzelne Angebote beziehen. Die Teilstudie ist bewusst offen gehalten, weil sich die Stichprobe relevanter mHealth-Angebote empirisch aus der Befragung ergibt. Dennoch sollte man im Vorfeld zumindest grob festlegen, (a) welche Arten von Inhalten analysiert werden sollen (z.B. Beiträge in Publikumsmedien, Fachmedien bzw. entsprechenden Portalen, Nutzerkommentare von Betroffenen) und (b) wie diese Beschreibungen und Bewertungen bei der notwendigen Aggregierung der Inhaltsanalysedaten pro mHealth-Angebot gewichtet werden.
Insgesamt überzeugt der Antrag theoretisch, konzeptionell, methodisch sowie in seiner Budgetplanung. Besonders erfreulich ist es, dass mit Nariman Sawalha die Mitautorin der Meta-Analyse für die Doktorandenstelle zur Verfügung steht und damit eine Person, die mit dem Themenfeld bestens vertraut ist. Die Begutachtungsgruppe empfiehlt die Förderung im beantragten Umfang.

Stellungnahmen zum Antrag

Forschungsgruppe: Digitale Medien im Selbstmanagement chronischer Erkrankungen (DISELMA)“ (FOR 5303)
Projekt: „Gesundheitsversorgung: Die Arzt-Patienten-Beziehung im Prozess der Übernahme und Alltagsintegration digitaler Medien für das Selbstmanagement chronischer Krankheiten“ (RI 2578/4-1)

Antragstellende Person: Frau Dr. Claudia Riesmeyer, München

Stellungnahme der Begutachtungsgruppe:

Die Antragstellerin will auf der theoretischen Basis von Akteurs-Struktur-Dynamiken untersuchen, wie Menschen im Alltag des digitalen Selbstmanagements die Beziehungen zu Ärzten gestalten. Gefragt wird, wie die Beziehung konzeptualisiert werden kann, wie sie ganzheitlich verstanden werden kann und wie die gegenseitige Wahrnehmung ist. Negotiation und mediation sind die zentralen Konzepte, die innerhalb der Interaktion untersucht werden. Die Beziehung wird als solche konzeptualisiert, wobei auf Akteurs-Struktur-Dynamiken Bezug genommen wird.
Als Forschungsdesiderate werden identifiziert (1) die Wahrnehmung der Ärzte und der Abgleich von Negotiation und Mediation mit den Ansprüchen des deliberativen Ansatzes (hier models) sowie (2) die Konzeptualisierung der Arzt-Patienten-Beziehung mithilfe der Akteurs-Struktur-Dynamiken und (3) die Erarbeitung einer holistischen statt einer selektiven Perspektive. Theorieziel ist es, die Ideen der Akteurs-Struktur-Dynamiken auf die Gesundheitskommunikation zu beziehen. Das wird im Antrag weitgehend überzeugend entwickelt. Die Forschungsziele sind differenziert, bleiben aber deskriptiv, was angesichts der neuen Perspektive nachvollziehbar ist. Um die Fragen zu bearbeiten, werden quantitative (250 Ärzte) Interviews, qualitative, als Panel-Studie angelegte Interviews und Beobachtungen (60 Dyaden) sowie zwei Focusgroups durchgeführt. Der Feldzugang soll mit relativ hohen Incentives gesichert werden.
Positiv hervorzuheben ist die Tatsache, dass die Interaktionen im Mikro-Bereich hier als Quasi-Meso-Ebene verstanden werden können. Das ist zwar im Teilprojekt selbst wenig ausgearbeitet, unterstützt aber eine zentrale Stärke des Gesamtprojekts, nämlich den Fokus auf die Meso-Ebene der Kommunikation zwischen Einzelnen in unterschiedlichen Rollen. Die kommunikativen Interaktionen spielen eine wichtige Rolle, auch im Selbstmanagement via digitale Medien. Das Modell der Beziehung ist überzeugend, lässt allerdings die Unterscheidung von Negotiation und Mediation sowie Fragen nach den Einflussfaktoren außen vor. Die Wahl der Methoden erscheint zielführend, bei der teilnehmenden Beobachtung und den Dyaden dürfte der Feldzugang trotz der Incentives herausfordernd sein.
Dier Forschungsüberblick ist gründlich und klar verständlich, die Forschungsdesiderata werden überzeugend begründet. Auch das Arbeits- und Fragenprogramm ist sinnvoll und passt perfekt in die Forschungsgruppe. Interessante Befunde verspricht v.a. eine stärkere Fokussierung auf die Wahrnehmungen der Behandelnden (im Gegensatz zur bisherigen Forschung, die sich hauptsächlich mit den Wahrnehmungen der Behandelten befasst hat) sowie die theoretische Fundierung auf Akteur-Struktur-Dynamiken nach Schimank. Kleinere konzeptionelle Fragen konnten in der Diskussion geklärt werden. Die qualitativ-quantitative Mehrmethoden-Ansatz ist überzeugend dargestellt. Besonders interessant findet die Begutachtungsgruppe die geplanten Leitfadeninterviews und -beobachtungen von Arzt-Patienten-Dyaden. Insgesamt überzeugt der Antrag theoretisch, konzeptionell, methodisch sowie in seiner Budgetplanung. Die Begutachtungsgruppe empfiehlt die Förderung im beantragten Umfang.

Stellungnahmen zum Antrag

Forschungsgruppe: Digitale Medien im Selbstmanagement chronischer Erkrankungen (DISELMA)“ (FOR 5303)
Projekt: „TP5: Der Einfluss informeller Netzwerkkontakte auf die Alltagsintegration digitaler Medien für das Selbstmanagement chronischer Erkrankungen“ (RE 3650/9-1)

Antragstellende Person: Frau Professorin Dr. Doreen Reifegerste, Bielefeld

---

<u>Stellungnahme der Begutachtungsgruppe:</u>

Dieses Vorhaben stellt ein wichtiges Projekt für die Forschungsgruppe dar. Die fachliche Kompetenz der Antragstellerin steht außer Frage; sie ist eine bestens ausgewiesene Expertin auf ihrem Gebiet.
Es ist sehr zu begrüßen, dass die Bedeutung sozialer Kontakte in der Gesundheitskommunikation analysiert werden soll. So ist der Antragstellerin auch zuzustimmen, wenn sie schreibt: „Consequently, a detailed look at the meso-level of social contacts is needed to explain why and how people integrate health-related digital media in their everyday life.“ (p. 1). Auch der Fokus auf informelle Kontakte ist in besonderem Maße sinnvoll. Wir reden eben viel mit Freunden oder Familienangehörigen über unsere gesundheitlichen Probleme und es ist nicht davon auszugehen, dass diese Kommunikation folgenlos bleibt. Positiv hervorzugeben ist zudem die systematische Unterscheidung in *informal contacts*, *formal contacts*, *institutional contacts*, und *media contacts*. Diese Strukturierung ermöglicht es, unterschiedliche Charakteristika der einzelnen Gruppen zu identifizieren und deren Einfluss auf die Implementierung und Nutzung digitaler Medien zum Gesundheits-Selbstmanagement zu beschreiben.
Gleichwohl fehlten der Begutachtungsgruppe in dem Antrag etwas spezifischere Begründungen, warum man dies untersuchen will. So bleibt es doch sehr im Allgemeinen, wenn man als Forschungsziel angibt, man wolle einen Beitrag leisten „(…) to the overarching question of the role of digital media in the self-management of chronic conditions, especially on the interpersonal level“ (p. 9).
Die Antragstellerin bezieht sich auf unterschiedliche medienbezogene theoretische Ansätze aus den Bereichen Diffusionsforschung, soziale Netzwerke und Every Day Life. Diese verschiedenen Ansätze und Themengebiete stehen noch etwas unverbunden nebeneinander. Eine eigenständige theoretische Perspektive der Antragstellerin ist wünschenswert. Man könnte auch fragen, ob es wirklich notwendig ist, sowohl den Diffussionsprozess als auch den alltäglichen Gebrauch von Gesundheits-Apps zu analysieren. Möglicherweise wäre der Fokus auf eines der beiden Themengebiete am Ende fruchtbarer.
Insgesamt stellt der Antrag gleichwohl konzise und ausführlich die Forschungslücken und den zu erwartenden Mehrwert sowie die wissenschaftliche und gesellschaftliche Relevanz des Projektes dar. Das Projekt ist für den innovativen Fokus des Gesamtprojekts auf die kommunikative Meso-Ebene existenziell. Die durch die Netzwerkanalyse gewonnen Erkenntnisse werden sowohl für die Mikroebene (TP 1-2) als auch die Makroebene (TP 6-7) von zentraler Bedeutung sein. Die Begutachtungsgruppe empfiehlt daher die Förderung im beantragten Umfang.

Stellungnahmen zum Antrag

Forschungsgruppe: Digitale Medien im Selbstmanagement chronischer Erkrankungen (DISELMA)" (FOR 5303)
Projekt: „Organisationale Kommunikation über die Nutzung digitaler Medien für das Selbstmanagement chronischer Erkrankungen (TP6: Organisationen)" (RA 1058/5-1)

Antragstellende Person: Frau Professorin Dr. Juliana Raupp, Berlin

---

Stellungnahme der Begutachtungsgruppe:

Der Antrag nimmt eine klassische Meso-Perspektive ein, fragt also nach der Sicht und dem Handeln der beteiligten Organisationen in Bezug auf die Nutzung digitaler Medien für das Selbstmanagement chronischer Erkrankungen. Das umfasst App-Anbieter, Pharmaindustrie, Versicherungen, Regulierer, Verbände. Diese Vielfalt an Organisationsformen und Interessen will die Antragstellerin auf ihre Kommunikations- und Informationsstrategien hin untersuchen. Hier soll eine Typologie entwickelt werden. Dabei wird das spezifische Framing in den unterschiedlichen Typen von Organisationen und vor dem Hintergrund unterschiedlicher Strategien untersucht, die Motive sowie die verbreiteten Inhalte.
Das Theoriegerüst ist neben dem allgemeinen Bereich der Organisationskommunikation der Neo-Institutionalismus und strategisches Framing. Unterschiedliche organisationale Logiken werden ebenfalls in den Blick genommen. Die bestehende Lücke einer Theorie der Organisationskommunikation für den Gesundheitssektor soll geschlossen werden, indem der Neo-Institutionalismus auf den Gesundheitssektor angewandt wird. Dazu wird auf Framing-Ansätze zurückgegriffen.
Das empirische Vorhaben umfasst eine Bestandsaufnahme und Typologie von Organisationen im untersuchten Bereich, 20 Experten-Befragungen von Kommunikationsverantwortlichen sowie Inhaltsanalysen mit Framinganalysen (Inhaltsanalysen, die dann via Clustering verarbeitet werden) von Apps und strategischer Kommunikation. Das Untersuchungsmodell illustriert die Komplexität des Feldes und macht klar, dass auch Netzwerkansätze hier eine wichtige Rolle spielen. Die Berücksichtigung der Meso-Perspektive ist ein großer Pluspunkt der Studie in der Gesamtarchitektur der Forschungsgruppe. Die Meso-Perspektive öffnet Möglichkeiten für Verbindungen Richtung Gesellschaft und Richtung Individuum. Die Vielfalt widerstreitender Interessen ist ein guter Anknüpfungspunkt für die Erklärungssuche für unterschiedliche Strategien in Bezug auf die Rolle von digitalen Medien beim Selbstmanagement.
In Bezug auf die Forschungsstrategie scheinen der Begutachtungsgruppe die Erklärungen für bestimmtes Verhalten der Organisationen etwas kurz zu kommen gegenüber den geplanten Bestandsaufnahmen und Beschreibungen. Dabei wären die Verbindungen zur Makro-Ebene und zur Mikro-Ebene des individuellen Managements vielversprechend.
Die Antragstellerin ist bestens ausgewiesen für die Analyse von Organisationskommunikation und Framing. Ihr Publikationsoutput ist sehr gut und ihre Projekterfahrung weit überdurchschnittlich.
Im Ergebnis handelt es sich um ein sehr überzeugendes Projekt, das eine zentrale Rolle für das Gesamtvorhaben spielt. Die Begutachtungsgruppe empfiehlt die Förderung im beantragten Umfang – lediglich die beantragten Hilfskraftmittel sollten auf 50.000 Euro gekürzt werden.

Stellungnahmen zum Antrag

Forschungsgruppe: Digitale Medien im Selbstmanagement chronischer Erkrankungen (DISELMA)“ (FOR 5303)
Projekt: „Medienberichterstattung über die Nutzung digitaler Medien für das Selbstmanagement chronischer Krankheiten (TP7: Berichterstattung)“ (ME 4745/1-1)

Antragstellende Person: Frau Professorin Dr. Julia Metag, Münster

---

Stellungnahme der Begutachtungsgruppe:

Das Projekt zielt auf eine Erfassung der massenmedialen Frames sowie der generellen Auswahllogiken bei der Berichterstattung (Nachrichtenfaktoren) zur Rolle digitaler Medien beim Selbstmanagement chronisch Kranker ab. Die Berichterstattung soll in Bezug auf den Anteil themenspezifischer Elemente (chronische Krankheiten sowie digitale Medien zum Management) sowie die Muster von Themen, Ereignismerkmalen und wissenschaftlicher Evidenz untersucht werden, die sich dort finden lassen. Untersucht werden soll die journalistische tagesaktuelle oder auch wochenaktuelle Berichterstattung sowie Zeitschriften und Spezialmedien zur Gesundheit. Geplant ist eine automatisierte Inhaltsanalyse für die eher allgemeine Analyse der Berichterstattung zu chronischen Krankheiten und eine zusätzliche manuelle Codierung für die Nachrichtenfaktoren und die Frameelemente zur Rolle digitaler Medien beim Selbstmanagement chronischer Krankheiten (per Clusteranalyse zu Frames konstruiert). Die Befunde lassen sich später in Beziehung setzen zu den Befunden in der strategischen Kommunikation.
Als Forschungslücken werden Studien zum Nachrichtenwert von chronischen Krankheiten, zur Berichterstattung über digitale Medien im Allgemeinen und ihrer Rolle bei chronischen Krankheiten im Besonderen, zur Berichterstattung über wissenschaftliche Evidenz beim Umgang mit digitalen Medien, sowie bei der Berücksichtigung von Zeitschriften und Spezialmedien ausgemacht. Entsprechend soll gefragt werden, wie Medien über die Nutzung digitaler Medien beim Selbstmanagement chronischer Krankheiten berichten.
Der Forschungsstand ist sehr umfassend und sorgfältig aufgearbeitet. Eine Stärke dieses Teilprojekts ist die systematische und methodisch anspruchsvolle Erfassung der Massenmedienberichterstattung zum Thema. Diese erlaubt eine relationale Bestimmung der Relevanzzuweisung zu chronischen Krankheiten und dem Einsatz digitaler Medien über die Zeit. Ferner können so inhaltliche Muster (Nachrichtenfaktoren, Frames und wissenschaftliche Evidenz) und ihre Veränderungen beobachtet werden. Umgekehrt könnten organisationale Interessen als Ursachen für veränderte Berichterstattung untersucht werden oder auch Wirkungen der Berichterstattung auf Kranke.
Die Antragstellerin hat ihren Schwerpunkt im Bereich der Wissenschaftskommunikation, v.a. der Berichterstattung über Technologie und Umweltfragen, und des Framing. Sie ist ferner gut vertraut mit automatisierter Inhaltsanalyse. Ihr Publikationsoutput ist beachtlich. Die Antragstellerin verfügt über umfangreiche Erfahrung im Bereich der Durchführung von wissenschaftlichen Projekten, mit einem Fokus auf Inhaltsanalysen.
Im Ergebnis handelt es sich um ein sehr überzeugend und gelungen dargestelltes Projekt. Ein sehr guter Antrag, der sehr stringent und sehr klar argumentiert. Die Kombination von automatisierten und manuellen IA ist sehr sinnvoll. Der Zeitplan erscheint sehr realistisch. Die Begutachtungsgruppe empfiehlt die Förderung im beantragten Umfang – lediglich die beantragten Mittel für Geräte und Software in Höhe von 7.000 Euro sollten gestrichen werden.
